# Electromagnetic Shielding by MXene-Graphene-PVDF Composite with Hydrophobic, Lightweight and Flexible Graphene Coated Fabric

**DOI:** 10.3390/ma11101803

**Published:** 2018-09-22

**Authors:** Kanthasamy Raagulan, Ramanaskanda Braveenth, Hee Jung Jang, Yun Seon Lee, Cheol-Min Yang, Bo Mi Kim, Jai Jung Moon, Kyu Yun Chai

**Affiliations:** 1 Division of Bio-Nanochemistry, College of Natural Sciences, Wonkwang University, Iksan 570-749, Korea; raagulan@live.com (K.R.); braveenth.czbt@gmail.com (R.B.); softaqua88@daum.net (H.J.J.); 2 Multifunctional Structural Composite Research Center, Institute of Advanced Composite Materials, Korea Institute of Science and Technology, Chudong-ro 92, Bongdong-eup,Wanju-gun, Jeollabukdo 55324, Korea; t14225@kist.re.kr; 3 Department of Chemical Engineering, Wonkwang University, Iksan 570-749, Korea; 123456@wku.ac.kr; 4 Clean & Science Co., Ltd., Jeongeup 3 Industrial Complex 15BL, 67, 3sandan 3-gil, Buk-myeon 56136, Jeongeup-si, Korea; jjmoon@cands.kr

**Keywords:** graphene, MXene, EMI shielding, composite, fabric

## Abstract

MXene and graphene based thin, flexible and low-density composite were prepared by cost effective spray coating and solvent casting method. The fabricated composite was characterized using Raman spectroscopy, X-ray diffraction (XRD), scanning electron microscope (SEM), X-ray photoelectron spectroscopy (XPS) and energy dispersive X-ray (EDX). The prepared composites showed hydrophobic nature with higher contact angle of 126°, −43 mN·m^−1^ wetting energy, −116 mN·m^−1^ spreading Coefficient and 30 mN·m^−1^ lowest work of adhesion. The composites displayed excellent conductivity of 13.68 S·cm^−1^ with 3.1 Ω·sq^−1^ lowest sheet resistance. All the composites showed an outstanding thermal stability and constrain highest weight lost until 400 °C. The MXene-graphene foam exhibited excellent EMI shielding of 53.8 dB (99.999%) with reflection of 13.10 dB and absorption of 43.38 dB in 8–12.4 GHz. The single coated carbon fabric displayed outstanding absolute shielding effectiveness of 35,369.82 dB·cm^2^·g^−1^. The above results lead perspective applications such as aeronautics, radars, air travels, mobile phones, handy electronics and military applications.

## 1. Introduction

The rapid advancement in intricate packing of modern electronic systems causes undesirable radiation; this inevitable radiation is known as electromagnetic interference (EMI), which has negative effects on humans and neighboring electronic systems. EMI pollution causes health hazards such as languidness, insomnia, nervousness, and headaches [1,2,3,4]. Electromagnetic compatibility can be achieved by using various materials such as textiles, polymer-based composites, MXene, and fabrics. EMI shielding is expressed in decibels (dB) [5,6,7,8,9,10,11,12,13,14,15]. Conductive and nonconductive polymers such as poly-p-phenylene-benzobisthiazole (PBT) [1,4,5], polythiophene (PTh) [1], Polyvinylidene fluoride (PVDF) [7,8,13], polyacrylic acid (PAA) [1], styrene polymethyl methacrylate (SPMMA) [4,5], and fillers such as metal nanoparticles [14,15,16,17,18], magnetic materials [13,14], carbon black, graphite [11], carbon nanotubes [9,10,11,12], graphene (GN) [19], and carbon fibers (CF) [17] are used to tune the properties of EMI shielding materials [20]. The polymer Nano composites (PNC) are widely used as advance engineering material in different environment. The functional materials, molecular dynamics, molecular details and micro structure of PNC are important for the application [21]. PNC consist Nano fillers play important role in generating conductive networks and combination of components alter the physicochemical properties of the composites [22,23,24,25]. Further, surface properties of the materials can be transformed in to hydrophobic/lyophilic by coating nanoparticle such as TiO_2_, ZnO and silica aerogel or polymers like polydimethylsiloxane (PDMS), polytetraflouroethylene (PTFE). The cross link/hydrogen bond between constituents cause by surface functional groups. The cross links improve the thermo mechanical properties [26,27,28]. Furthermore, in the polymer foams the voids form due to the different nucleation time of constitutional solid and other external factor like temperature pressure [29]. Advanced EMI shielding materials should be lightweight, flexible, cost effective, dielectric, and multifunctional, and should possess a tunable absorption, high thermal resistance, intrinsic conductivity, large aspect ratio, high corrosion resistance, and good magnetic and electronic properties [19,20,30,31,32,33,34].

Recently, flexible, corrosion resistant, high-density, thin carbon-based materials with satisfactory electrical conductance have become attractive candidates for EMI shielding applications such as in the aerospace, aircraft, automobile, and modern electronics fields. Hence, wet-laid synthetic nonwoven fabrics fulfil these criteria with good EMI shielding [34]. In addition, carbon-carbon-based composites possess greater EMI shielding effectiveness than carbon-based polymer matrices. Further, continuous carbon fibers are preferred to discontinuous fibers in carbon-based EMI shielding materials [35]. This is because the properties of carbon fiber that affect EMI shielding those are the length and array [36]. Further, MXene resembles graphene, is an attractive engineering material and used as filler exploited to create flexible electronic devices and other engineering materials [37]. The EMI shielding range of most graphene/PVDF composites of various thicknesses has been reported to be in the range of 20–30 dB. In addition, the graphene can be functionalized by using reduction, oxidation, metal nanoparticles, organic molecules and polymers for various applications like solar cell, antibacterial materials and the EMI shielding of graphene/PVDF has been enhanced by the decoration of nanoparticles [38,39,40,41,42].

Two-dimensional MXenes are explored intensively for various applications including EMI shielding. MXenes are sprouting transition metal (Ti, V, Cr, Nb, and Ta) carbides/nitrides with universal formula M_n+1_X_n_T_x_ (n = 1, 2, and 3), where M is an early transition metal, X is carbon or nitride, and Tx is a surface functional group (−O, =O and F). MXenes are generated from the corresponding layered MAX phase with the general formula M_n+1_AX_n_ by selective engraving of the A-layer (group 13/14 elements) created by a weak M-A bond sandwiched between a strong M-X bond. Minimally intensive layer-delamination (MILD) etching is carried out using the LiF/HCl method, which is advantageous over clay etching in which Hydrogen fluoride (HF) is utilized under various etching conditions [43,44,45,46,47]. Intercalation and exfoliation are conducted using urea, dimethyl sulfoxide (DMSO), tetramethylammonium hydroxide (TMAOH), NH_4_OH, tetrabutylammonium hydroxide (TBAOH), and sonication. These exfoliation techniques are inevitable in the clay method. However, LiF/HCl-based in-situ mild etching is highly preferable owing to the number of steps, level of defects and risk, and the fact that exfoliation can be achieved through manual shaking. However, sonication at low temperature and in inert environments (Ar) is preferable [34]. MXene thin-films and foams exhibit the highest EMI shielding in the X-band region. EMI shielding can be achieved by absorption, reflection, and multiple reflection. The MXene film enables internal multiple reflection which facilitates absorption. The reflection on the surface due to the electron and layered structure encourages multiple reflection. When electromagnetic radiation hits the surface, it induces electron mobility (ohmic loss). The lightweight foaming materials are attractive candidate over metal-shielding materials as the latter have higher densities which limit the application range in terms of aerospace [34,35].

In this study, we develop a graphene-flake (GN) coated carbon-fiber reinforced-matrix composite (MC) and solution-casting MXene graphene foam, which exhibit a high EMI shielding effect in the S-band region. The required thickness is achievable by adjusting the spraying and drying cycles. Further, we developed MXene graphene foam with internal hollow sphere with surface imbedded balls. Consequently, we analyze the following parameters in detail; EMI shielding, morphology of GN-coated matrix and MXene-graphene foam, electrical conductivity, constitutional chemical species, elemental percentage, and hydrophobic nature. In addition, the pristine carbon-fiber-reinforced matrix composite, graphene, graphene oxide, and reduced graphene oxide are denoted as MC, GN, GNO, and rGNO, respectively. The GN, GNO and rGNO coated fabrics are denoted as GNMC, GNOMC, and rGNOMC, whereas the MXene-graphene coated fabric, MXene-graphene composite, and MXene-graphene oxide composite are symbolized as MGNMC, MGNC, and MGNOC, respectively.

## 2. Materials and Methods

### 2.1. Materials

Graphene (GN) (M-25, 99.5%, average size and thickness of 25 µm and 7 nm, respectively) was obtained from Ditto Technology Co. Ltd., (Gyeonggi-do, Seoul, Korea). Dimethylformamide (DMF) 99.8 *w*/*w*%, lithium fluoride (LiF) (98%, 300 mesh), Polyacrylic acid (PAA), and Polyacrylamide (PAM) were purchased from Sigma Aldrich (Seoul, Korea). Polyvinylidene fluoride (PVDF) (melting point of 155–166 °C) was purchased from Alfa Aesar (Seoul, Korea). Hydrochloric acid (HCl-35%) and nitric acid (HNO_3_-70%) were supplied by Samsung Chemical Co., Ltd. (Seoul, Korea), anhydrous lithium chloride (LiCl) was purchased from Tokyo Chemical Industry Co., Ltd (Tokyo, Japan), and Ti_3_AlC_2_ was acquired from Forsman Scientific Co., Ltd. (Beijing, China). Carbon fiber (fiber diameter 7 µm, 6 mm) and polyethylene terephthalate (PET) binder (fiber diameter 2.2 dtex, 5 mm) were purchased from TORAY Product (Osaka, Japan). No purification methods other than those stated were utilized for the chemicals.

### 2.2. Preparation of Graphene Oxide (GNO) and Reduced Graphene Oxide (rGNO)

A total of 1 g of graphene was mixed with 50 mL of HNO_3_ and stirred at room temperature for 12 h. The reacted graphene was washed with deionized water until it reached a neutral pH. The resulting black flakes were GNO, and these were dried at 80 °C for 24 h. Equal amounts of GNO and NaBH_4_ were mixed together in deionized water and stirred at room temperature for 12 h. The resultant product was washed several times with deionized (DI) water and dried at 80 °C for 24 h. The obtained product was rGNO.

### 2.3. Preparation of MXene and MXene Colloidal Solution

Equal amounts of Ti_3_AlC_2_ and LiF were immersed in 20 mL of 6M HCl solution and stirred at 35 °C for 24 h. The resultant mixture was washed with DI water (pH 6) several times by centrifuging at 3500 rpm for 5 min, and the black flakes were dried at 100 °C for 12 h in a vacuum oven. A total of 0.1 g of MXene was dispersed in 10 mL of DI water by sonication for 1 h in an ice bath. The resultant exfoliated solution was centrifuged at 3500 rpm for 30 min. The supernatant was collected and stored at 5 °C for the coating process.

### 2.4. Preparation of Carbon Fabric

Carbon fiber, PET-binder fiber with a 4:1 weight ratio, and 0.3 wt.% of PAM were dispersed in DI water. Then, the mixture was rotated at 500 rpm for 10 min. A web was produced using a general wet-laid method. During this process, a drum dryer was used with a surface temperature of 140 °C and a speed of 7 m·min^−1^. The obtained fabric density was 20 g·m^−2^.

### 2.5. Fabrication of Composite (MC)

A series of GN-coated MCs were prepared by a cost-effective spray-coating process. MC was spray-coated using 3 g·L^−1^ of GN, GNO, and rGNO with a 5 g·L^−1^ PVDF dispersed solution of DMF. After the coating process, the fabrics were subjected to drying at 100 °C for 5 min in a drying oven. This process was repeated up to ten cycles to alter the quantity of GN coated on the MC in each case. MNNC and MGNOC were fabricated using a solvent-casting method; 5 g of PVDF, 3 g of GN, and equal amounts of PAA and LiCl (0.3 g) were stirred in a 50 mL DMF solution at room temperature for 12 h. The resultant mixture was poured into a casting plate and evaporate DMF in vacuum oven at 80 °C (pressure below 0.8 atm). Then, 100 mL of colloidal MXene solution was added and evaporation occurred under the same condition. Finally, the resultant film was separated from the casting plate.

### 2.6. Characterization

The density was measured using a laser flash apparatus, LFA457 (NETZCH, Seoul, Korea). A high-resolution Raman spectrophotometer Jobin Yvon, LabRam HR Evolution (Horiba, Tokyo, Japan) was used to identify the structural features of MC, GN, GNO, rGNO, MXene, and GN-based and MXene composite. The morphologies of the fabrics were investigated using a field-emission scanning electron microscope (SEM, S-4800; Hitachi, Tokyo, Japan). The X-ray diffraction patterns of the materials were recorded using a high-power X-ray diffractometer, D/max-2500V/PC (Ragaku, Tokyo, Japan) with Cu (Kα). The elemental percentages and chemical environments were analyzed using XPS with a spot-size of 30–400 µm at 100 W of Emax (Al anode) K-Alpha, Thermo Fisher (East Grinstead, UK). A contact angle meter, Phonix-300A (S.E.O. Co., Ltd., Suwon, Korea), was used to analyze the wetting ability of the surfaces of the composites. A thermal analyzer, DSC TMA Q400 (TA Instruments Ltd., New Castle, DE, USA), was used to measure the thermogravimetric data. The EMI shielding effectiveness (SE) of the composites were recorded using an EMI shielding tent, ASTM-D4935-10, ASTM International (West Kentucky, PA, USA) at room temperature (For s band). The Savitzky–Golay function (Origin 2017 graphing and analysis, OriginLab; Boston, MA, USA) was used to plot the data. The electrical conductivities were measured using a four-probe method FPP-RS8, DASOL ENG (Seoul, Korea). The thicknesses were measured using a Mitutoyo thickness 2046S dial gage (Mitutoyo, Kanagawa, Japan). The electromagnetic characteristics of the specimens were measured using a vector network analyzer (VNA, Agilent N5230A, Agilent Technologies, Santa Clara, CA, USA) and a rectangular wave guide with the frequency ranging from 8.2 GHz to 12.4 GHz. The samples were prepared by cutting the free-standing film into rectangular shapes (width is 22.16 mm and height is 10.16 mm) (For X band).

## 3. Results

### 3.1. Structural Characterization

#### 3.1.1. Scanning Electron Microscopic (SEM) Analysis of Morphology

**Figure 1:**
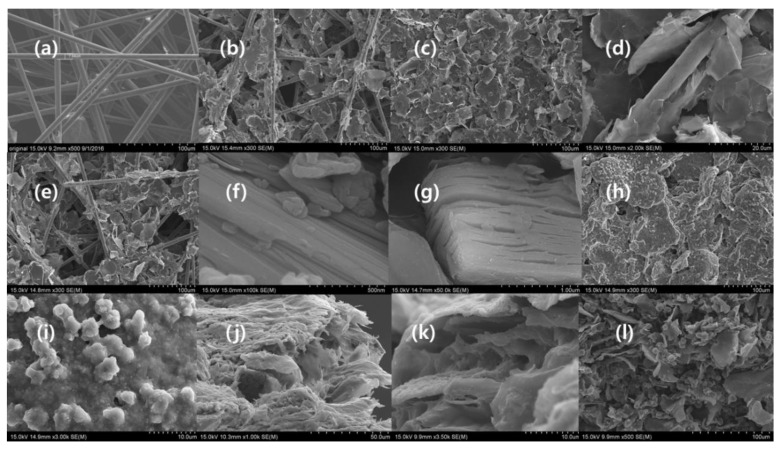
Microstructural images from scanning electron microscopy of (**a**) surface of MC (×500), (**b**) surface of GNMC (×300), (**c**) surface of GNOMC (×300), (**d**) fiber surface of GNO-coated GNOMC (×2000), (**e**) surface of rGNOMC (×300), (**f**) Ti_3_AlC_2_ (×100,000), (**g**) Ti_3_C_2_T_x_ (×50,000), (**h**) surface of MGNC (×300), (**i**) MXene on surface of MGNC (×3000), (**j**) cross-section of MGNC (×1000), (**k**) cross-section of MGNC (×3500) (**l**) cross-section of MGNOC (×500).

SEM images were used to analyze the surface topological morphology of the Ti_3_AlC_2_, Ti_3_C_2_T_x_, graphene, MXene composites, and uncoated fabric (MC). Virtually the cracks and annular gaps are entailing with fiber surfaces of MC (Figure 1a,d). The SEM image of MC (Figure 1a) expresses the porous, smooth, and clean nature of the surfaces, which consist of haphazardly packed carbon fibers and GN, GNO, and rGNO. They are oriented randomly and grooves remain owing to the wrinkly nature of graphene (Figure 1b,e) [48]. GNO is disseminated planar in nature (rigid stack) over the MC composite, which exhibits a different pattern to GN and rGNO [49]. This phenomenon is attributed to the presence of carboxylic groups and the flat nature (Figure 1c) of the GNO regulated arrangement of the graphene flakes on MC. In addition, the relevantly sized GN flakes could fill the fissures during fabrication (Figure 1b–e). This could be described in terms of the magnitude of the GN flakes used, and the size of the carbon fibers and gaps present in the fabric. The diameter of the carbon fibers is approximately 7–9 µm, whereas the average size of the GN flakes is 25 µm. Thus, the large size of the GN flakes prevents homogeneous coating of the smaller carbon fiber in the carbon fabric, as shown in Figure 1b–e. As a result, the majority of the pores are covered by carbon flakes owing to infiltration in the carbon fabric while smaller GN flakes (2–5 µm) are deposited on the surface of the carbon fiber (Figure 1d). Aggregation of GN at the carbon-fiber (CF) joints was observed and is shown in Figure 1b,d,e; this may enhance the hydrophobicity, EMI shielding, and electrical conductivity. Hence, the porosity of MC was attenuated by the coating process (Figure 1b–e) and alignment of the GN flakes can be tuned by oxidation (Figure 1c). This appears to be true based on our study. Ti_3_AlC_2_ and Ti_3_C_2_T_x_ are layered materials that are like graphite (Figure 1f,g) [50]. The gaps in Ti_3_C_2_T_x_ (Figure 1g) indicate that effective eradication of Al, and EDX strengthens this statement (Figure S3b). The surface of MXene-graphene foam illustrates the arrangement of the GN flakes and MXene with small pores (Figure 1h–k), where one graphene flake accommodates several MXene flakes. This could be an effective way to enhance multiple reflection and absorption. Moreover, interconnected MXene and graphene are responsible for electron mobility. The cross-sections of MGNC and MGNOC confirm that the formation of the foam, which is a highly attractive structural requirement for lightweight EMI shielding (Figure 1j,k) [34,35]. The cross-sections of MGNC and MGNOC visually confirm the foam structure (Figure 1j–l). It is obvious that the pore size of MGNOC is smaller than that of MGNC. This can be explained by the thickness of the material. The thicknesses of GNMC, GNOMC, rGNOMC, MGNC, and MGNOC are 0.0191, 0.0174, 0.0163, 0.0192, 0.035, and 0.0243 cm, respectively. The thickness of MGNOC is smaller than that of MGNC, which means that the pores in MGNOC are small and GNO is arranged in a flat stack. Further, the cross section of coated fabric revealed the infiltration of GN, GNO, rGNO and MXene (Figure S2a–f). Most of the GNO flake laid on the surface of fabric while few penetrate (Figure S2c). The MGNC, MGNOC possessed internal hollow sphere with numerous ball like structure (Figure S2g,h). The size of the hollow sphere was large in MGNC whereas GNO densely packed with small spheres (Figure S2g,h). The EDX confirms the constitutional elements of Ti_3_AlC_2_ and Ti_3_C_2_T_x_ (Figure S3a,b) and that the etching removed Al and introduced F and Cl, derived from etching solution. The ratio F/O is 6.27 and F/Cl is 100.15, confirming that F is the major surface functional group. The mapping of the MGNC inveterate distribution of the elements in the composites are shown in Figure S3c–f.

#### 3.1.2. Raman Spectroscopic Analysis of the Structure of Carbon-Based Materials

**Figure 2:**
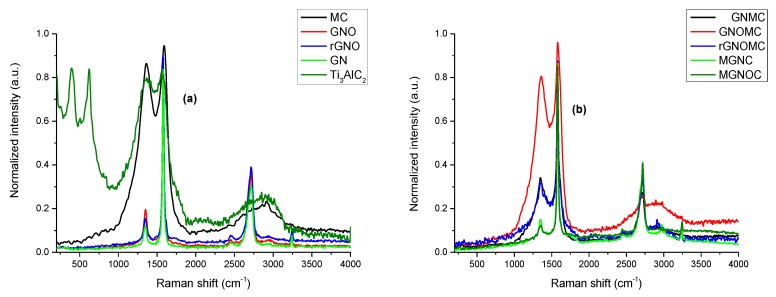
Normalized Raman spectra of (**a**) MXene, MC, GN, GNO, rGNO, and (**b**) composites.

Raman spectroscopy is a prominent tool with which to investigate the structural and crystalline nature of Ti_3_C_2_T_x_, and carbon-based materials including graphite materials [32,33]. In addition, the level of defect and disorder can be predicted by using (I_D_/I_G_) [6]. The I_D_/I_G_ value of GN, GNO, rGNO were 0.14, 0.23 and 0.17, respectively (Figure S4). Hence, oxidation made more defect in GNO while reduction minimize the defect rGNO. Furthermore, GNMC, GNOMC, rGNOMC, MGNC, MGNOC and MC had (I_D_/I_G_) value of 0.4, 0.84, 0.38, 0.17, 0.15 and 0.91 respectively. It was obvious that graphene coating diminished defects and films possessed less defects compare with fabric. MGNC foam consisted little high defect than MGNOC as MGNC own large hollow cavity than MGNOC (Figure S2). Introduction of hydroxyl functional groups lessen defect in carbon fabric while carboxylic acid group increase the defects [37]. Even though, carboxylic functional groups induced planer arrangement of graphene flake (Figure 1c). Further, the in-plane vibrational mode of surface functional groups Ti and C generate peaks at 624, 263, and 394 cm^−1^ [51,52]. The weak broad band with similar intensities at 1350 and 1570 cm^−1^ is attributed to the D- and G-bands. In addition, the presence of anatase TiO_2_ caused peaks at 628, 510, and 396 cm^−1^ (Figure 2b) [43,53]. The Raman spectra G-bands of GN, GNO, and rGNO show bands at 1578, 1580, and 1579 cm^−1^, respectively; these have higher intensities than the corresponding D-bands at 1351, 1352, and 1346 cm^−1^, respectively [51]. However, rGNO shows a weaker peak at 1346 cm^−1^ (Figure 2a). These results agree that GN- and GN-based materials have higher crystallinity. Highly oriented pyrolytic graphite (HOPG) is a form of ordered graphene (GN) sheets arranged one over another; the Raman spectrum of HOPG also manifests as a single band at 1582 cm^−1^ (G mode E2g) which corresponds to the band at 1578 cm^−1^ in the GN spectrum [32,54,55]. The raw material and production methods influence the disparity properties of carbon fiber, in which the constituents resemble graphite [56]. The Raman spectrum of MC exhibits numerous peaks, in which the D- and 2D-bands are placed at 1348–1374 cm^−1^ and 2680–2740 cm^−1^, respectively; these values are from the corresponding boundaries of CF crystalline graphite. In addition, the presence of HOPG is confirmed by the G-band at 1503–1634 cm^−1^ (Figure 2a) [32,33,56]. The use of PVDF as a binder in the GN coating influences the shape of the spectrum owing to the PVDF/GN interactions that cause fluctuation at 2750 cm^−1^ (2D-band), which is absent in MC. The bands in the spectrum split into a few new bands owing to the PVDF molecules [57,58]. In addition, GNOMC produces weak 2D band, whereas less oxidized composites exhibit a prominent 2D band. At the same time, the sharp band at 1503–1634 cm^−1^ and new peak at 2750 cm^−1^ provide evidence that the GN coating occurs on MC. In addition, the MGNC and MGNOC composites generate new peaks at 2452, 2976, and 3243 cm^−1^ while the G- and 2D-band intensities increase significantly. This advocates that effective interaction occurred between MXene, GN, and the polymers (Figure 2b).

#### 3.1.3. X-ray Diffraction (XRD) Analysis

**Figure 3:**
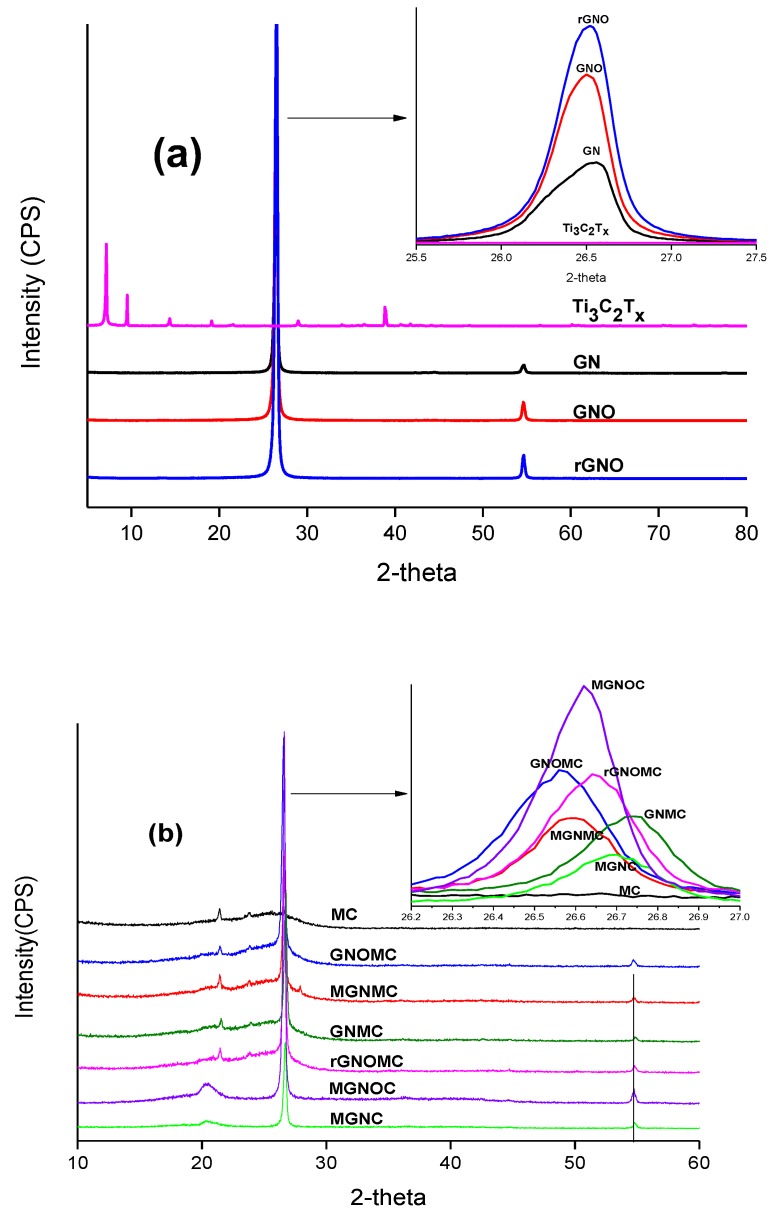
XRD of (**a**) rGNO, GNO, GN, MXene and (**b**) composites.

The crystalline or amorphous nature of the materials can be confirmed using XRD profiles [59,60]. XRD results of the pristine materials and composites are shown in Figure 3a,b. According to the XRD profiles, all of the materials display a crystalline nature. GN, GNO, and rGNO show two type of peaks: one intense peak 2θ located at 24.5°–27.5°, and another small peak 2θ positioned at 54.8°. However, the location of 2θ of the intense peak varies slightly such that 2θ is 26.56°, 26.5°, and 26.52°, which represent GN, GNO, and rGNO, respectively. The XRD pattern of Ti_3_C_2_T_x_ confirms the formation of MXene. Cao et al. reported that the delamination of MXene can be confirmed by the shifting of the peak from 9.3° to 7.2° [61,62]. Hence, synthesized MXene consisting of two peaks at 7.15° and 9.5° (002) confirm the formation of partially delaminated Ti_3_C_2_T_x_. The composites show three different peaks of 2θ = 19.5°–21.5°, 25.5°–27.2°, and ~54.8°. The high intense peaks are located at 2θ = 25.5°–27.2°, where MGNOC, GNOMC, rGNOMC, MGNMC, GNMC, and MGNC are positioned at 26.62°, 26.56°, 26.64°, 26.6°, 26.75°, and 26.7°, respectively. The intense peaks are attributed to the presence of graphene and PVDF [59,60]. In addition, the intense peak is absent in MC where the broader peak indicates the amorphous nature and presence of the graphite-like structure (Section 4.1.2, [63]). The peak at 2θ = 54.8 and 25.5°–27.2° confirms the presence of the graphene structure. In MGNMC, extra peaks are formed by MXene at 2θ = 23.8° and 27.9°. PVDF generates two weak shoulder 2θ peaks at 17.7° and 20.6° corresponding to alpha and beta PVDF, respectively [60]. MGNC and MGNOC display weak single peaks at 20.4°, which supports the peak due to PVDF. This peak is absent in the fabric-based composites owing to the low concentration of PVDF.

#### 3.1.4. X-ray Photoelectron Spectroscopy (XPS) Analysis

**Figure 4:**
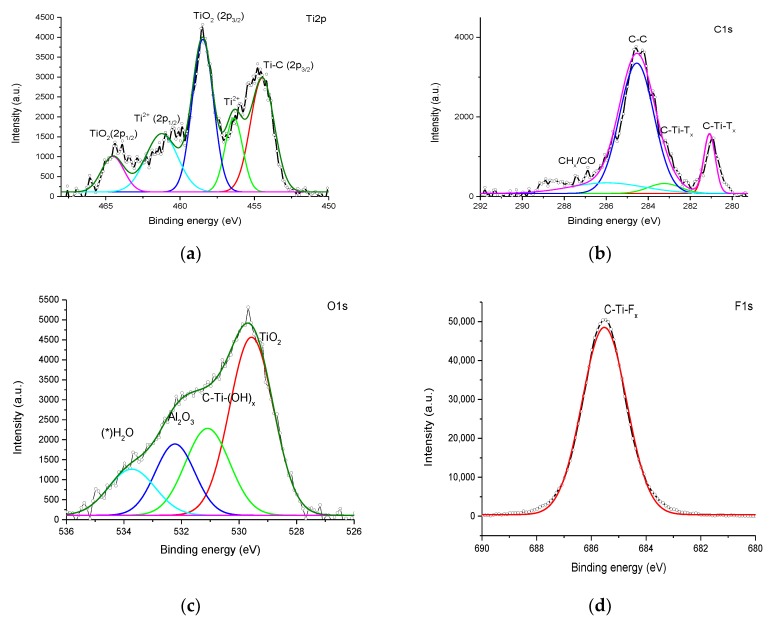

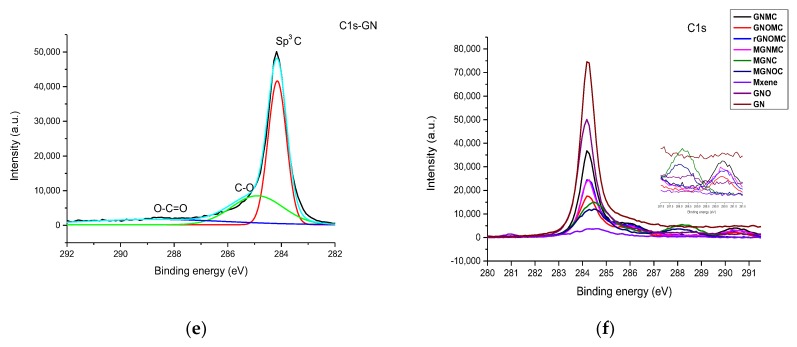
XPS fitting curves of Ti_3_C_2_T_x_ (**a**) Ti2p (**b**) C1s, (**c**) O1s, (**d**) F1s, and (**e**) fitting curve of the GN C1s; (**f**) overlapping curves of GNO, rGNO, GN, MXene, and graphene/ MXene-graphene composites.

XPS is useful technique that can deliver the structural nature and functional groups of the compound analyzed; a Gaussian–Lorentzian function is used to fit the XPS data. Thus, different binding energy levels were identified by using fitted Ti2p, C1s, F1s, and O1s electron binding energy curves. In addition, the bonding nature of diverse components is reported based on the chemical shift of elements (Figure 4a–f) [64,65]. Table 1 expresses the constitutional elements in different proportions. In MXene, F is a more dominant functional group than OH. The atomic percentage of the oxygen reveals slight oxidation of GN in GNO (Table 1). The XPS Ti2p fitting curve confirms the presence of bonds such as TiO_2_ (464.5(2p1/2) and 458.5 (2p3/2) eV), Ti^2+^ (461.3 and 456.4 eV), and Ti-C (454.5 eV). Further, C1s displays bonds such as C–Ti–Tx (281.1 and 283.2 eV), C–C (284.5 eV), and CHx/C=O (286.1 eV) where the C–C bond gives rise to a high intense peak. The functional constitutions, namely TiO_2_ (529.6 eV), C–Ti–(OH)x (531.1 eV), Al_2_O_3_ (532.3 eV), and H_2_O_ads_ (533.8 eV) are inveterate by the O1s fitting curve. The F1s fitting curve is purely responsible for the C–Ti–Fx bond. Hence, MXene is formed with the formula Ti_3_C_2_T_(OH, F)_ [52,66,67,68]. GNs comprise mainly graphene C-C bonds with numbers of C–O/C=O bonds. MC comprises 8.8% oxygen (Table 1); nevertheless, C=O or C–O belonging to the C1s peak are not observed prominently, which is confirmed by the C1s fitting curve of GN. However, the addition of PVDF introduces two main new peaks at 286 and 290.5 eV. These peaks might originate from the C–C–F and C–F bonds, respectively, and the 286 eV peak arises owing to the MXene C=O bond (Figure 4f). In addition, the newly generated MGNC and MGNOC peak at 288.1 eV may arise owing to the addition of PAA and LiCl [68]. However, the intense peak intensity and corresponding binding energy caused by the composites vary as follows: GNMC (284.17 eV), GNOMC and rGNOMC (284.25 eV), MGNMC (284.21 eV), and MGNC and MGNOC (284.5 eV) (Figure 4f). The XPS graphs of GN and other coated carbon composites show a combination of GN, PVDF, and carbon fabric peaks. The amount of O varies with the combination of the composite, which is strongly evidenced from the XPS data (Table 1). After the GN coating, we observed that there is defect at 285.0 eV, which may reduce the strength of the GNMC fabric.

### 3.2. Surface Property of Composites

The hydrophilicity associated with wettability plays a vital role in moistening the surfaces. A contact angle above 90° is considered hydrophobic, and below 90° is hydrophilic. Water-loving constitutions reduce the contact angle, whereas water-abhorring compounds increase the contact angle. The contact angle can be tuned by using organic or inorganic materials [69]. The spreading of the liquid on the surface depends on the surface energy between the solid and liquid. The increasing surface roughness and surface energy causes the hydrophobic nature [70]. When the roughness increases, the air is trapped in nano or micro grooves. This air minimizes the wetting area and leads to hydrophobicity. Hence, the topography of the materials and their other properties, such as morphology, roughness, and chemical homogeneity, influences the surface wettability [71]. The wetting ability of the composites are shown in Figure 4. GNMC, GNOMC, and rGNOMC exhibit a hydrophobic nature at 125°, 124°, and 126°, respectively, whereas MGNC and MGNOC show hydrophilic behavior at 78° and 81°, respectively. The wetting energies of GNMC, GNOMC, rGNOMC, MGNC, and MGNOC are −41.85, −41, −42.82, 14.89, and 11.48 mN·m^−1^, respectively. It is obvious that the positive wetting energy increases the hydrophilic nature. The most negative wetting energy (−42.82 mN·m^−1^) causes the highest contact angle and the contact angle is incommensurate with the wetting energy. The spreading coefficients of −114.65, −113.8, −115.62, −57.91, and −61.31 mN·m^−1^ were generated from GNMC, GNOMC, rGNOMC, MGNC, and MGNOC, respectively. The spreading coefficient also expresses a similar behavior to the wetting energy in terms of hydrophobic behavior. The rising work of adhesion increases the water-loving behavior, for instance, GNMC, GNOMC, rGNOMC, MGNC, and MGNOC engender values of 30.95, 31.8, 29.98, 87.69, and 84.28 mN·m^−1^, respectively; the increasing work of adhesion increases the hydrophilicity of the surface [69,70]. Hence, coating the graphene-based materials increases the hydrophobicity of the surfaces. Tissera et al. reported that GO-coated cotton showed an improvement in hydrophobicity with a maximum contact angle of 143° [72]. Zhang et al. reported that poly (vinylidene fluoride—hexafluoropropylene)/graphene composite is super hydrophobic in nature [73]. Despite this, the MXene-graphene-based foam exhibits a hydrophilic nature which is due to the surface MXene flakes. The produced composite can be used to protected instruments from harmful water environments.

### 3.3. Electrical Conductivity

**Figure 5:**
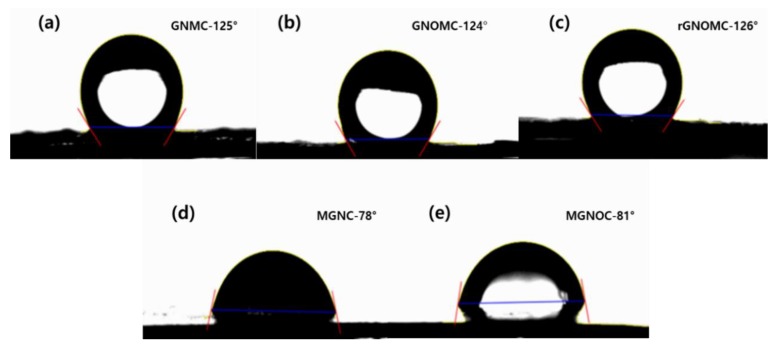
Contact angles of (**a**) GNMC, (**b**) GNOMC, (**c**) rGNOMC, (**d**) MGNC, and (**e**) MGNOC.

**Figure 6:**
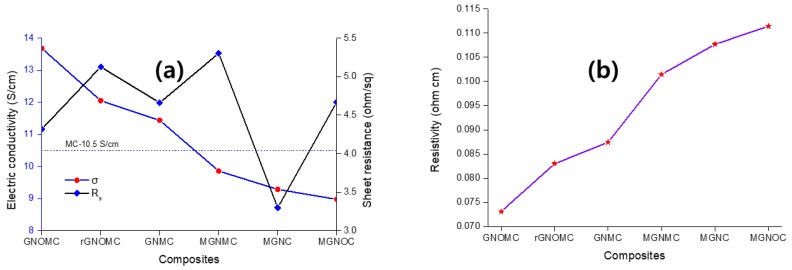
(**a**) Electric conductivity and sheet resistance of the composites and (**b**) resistivity of the composites (R_s_: sheet resistance; σ: electric conductivity).

The electrical conductivity of MC is significantly affected by the spray-coating process. The incorporation of 2D materials in the polymer alters the electric conductivity owing to the arrangement of the 2D material in the polymer matrix [74]. In graphene, the carbon atoms are arranged hexagonally with sp2 hybridization and the free π valance electron aligns at right angles to the hexagonal plane. This electron is responsible for the out-of-plane π bond and electron mobility. The conductivity of the graphene influences by the number of graphene layers. When the number of layers increases, the electrical conductivity reduces, which is due to the interfacial alignment of GN which increase the resistance [75]. GNOMC displays the highest electric conductivity of the composites, which is supported by the SEM image of GNOMC (Figure 1c). GNO arranges in a flat-stack manner with possible touching of the GNO flakes, which leads to interfacial electron transfer. Hence, etching with HNO_3_ is the best option to tune the self-assembly of GNO flakes on the MC matrix. The conductivity is inversely proportional to the thickness [76], and the conductivity and R_s_ of GNOMC are 13.68 S·cm^−1^ and 4.2 Ω·sq^−1^, respectively, at a thickness of 0.0174 cm (Figure 5). Nevertheless, GNOMC deviates from the MGNC behavior, exhibits a low electric conductivity (9.3 S·cm^−1^) while showing the lowest sheet resistance (3.1 Ω·sq^−1^) at a 0.0350 cm thickness; MGNOC exhibits 8.97 S·cm^−1^ with a 4.6 Ω·sq^−1^ sheet resistance and thickness of 0.0243 cm. Of the fabricated composite, MGNC shows a maximum thickness of 0.0350 cm, while the others, such as GNMC (0.0191 cm), GNOMC (0.0174 cm), rGNOMC (0.0163 cm), MGNMC (0.0192 cm), and MC (0.0127 cm) exhibit values below 0.0200 cm. Hence, the highest thickness of MGNC minimizes the electric conductivity. In addition, MGNMC shows the highest R_s_ value owing to the aggregation of the hydrophobic PVDF and hydrophilic MXene. The highest electrical mobility increases the EMI SE. Hence, the lowest sheet resistance of MGNC causes it to possess the highest surface electron mobility, which leads to the surface reflection of EMI SE [47]. Further, the resistivity of GNOMC, rGNOMC, GNMC, MGNMC, MGNC and MGNOC were 0.073, 0.083, 0.087, 0.101, 0.108 and 0.111 Ω·cm, respectively. Despite the conductivity depend on thickness of the materials. The functionalized graphene increased the resistivity while presence of MXene significantly increased the resistivity of fabric and foam (Figure 6b). Despite this, other parameters such as thickness and some other structural features (foams) also influence EMI SE [46]. Further, the lowest R_s_ and high resistivity of MGNC is due to the presence of MXene on the surface of the composite (Figure 1i). Further explanation is given in the EMI-shielding section.

### 3.4. Electromagnetic Shielding Effectiveness of Composites

**Figure 7:**
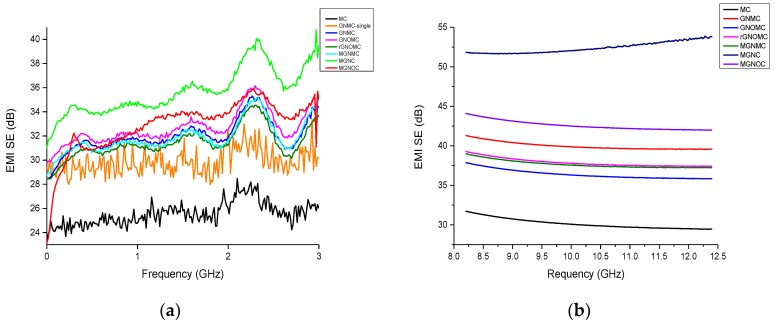

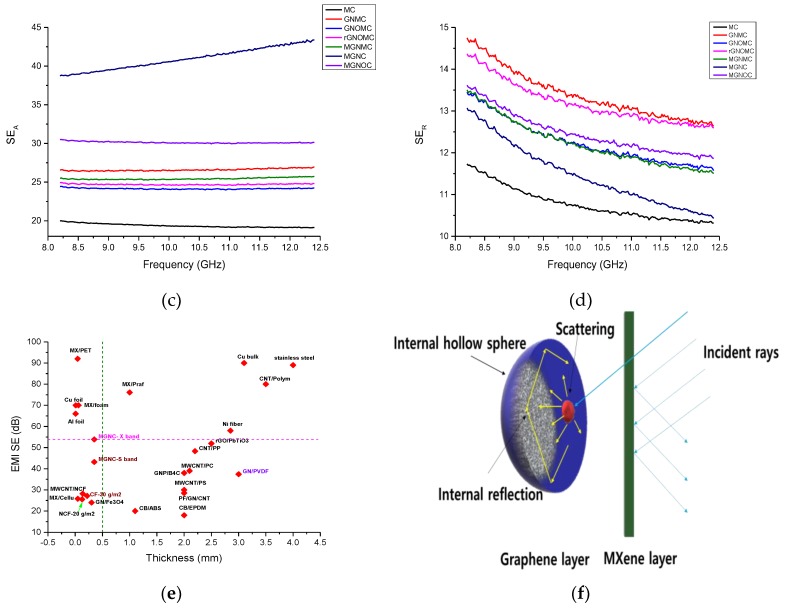
EMI shielding effectiveness of composites (**a**) EMI SE in S-band, (**b**) EMI SE in X-band, (**c**) SE_R_, (**d**) SE_A_, (**e**) comparison of EMI SE with thickness and (**f**) Basic mechanism in MGNC.

In this study, solution casting and spray coating were performed to produce EMI shielding composites. MC was spray-coated by a dispersed mixture of GN, GNO, and rGNO (3 g·L^−1^) and PVDF (5 g·L^−1^) in in a DMF solution. The thickness of MC was adjusted by changing the number of coating cycles. All of the EMI SE calculations were carried out according to the Gamage et al. study. The EMI shielding of all of the composites is illustrated in Figure 7. It is obvious that all of the composites show a maximum EMI SE in the frequency range of 1.9–2.6 GHz in S band region whereas GNMC showed increasing trend in X band region and other composites exhibited slight downward trend. Of the composites, MGNC yields the maximum and minimum EMI shielding of 41 and 31 dB, respectively, whereas MGNOC exhibits a 36 dB maximum and 23.14 dB minimum EMI shielding in S band region. The maximum EMI shielding of GNMC, GNOMC, rGNOMC, MGNMC, MC, and GNMC-single are 35.3, 36.2, 34.6, 35.2, 28.5, and 33.4 dB, respectively, and the corresponding minimum EMI shielding is 28.4, 29.7, 28.4, 28.8, and 23.2, 28 dB, respectively. The average EMI shielding of GNMC, GNMC-single, GNOMC, rGNOMC, MGNMC, MGNC, and MGNOC is 32, 30, 32.66, 31.43, 31.87, 35.7, and 32.86 dB, respectively in S band region. This trend changed in X band region that can be represented as follow, the maximum EMI shielding of GNMC 53.89 dB with reflection of 13.10 dB and absorption of 43.38 dB (Figure 7 and Table S2). The maximum EMI SE range of composites was 53.89–31.73 dB while minimum range was 52.4–30.15 dB (Table S2). The maximum reflection loss (SE_R_) and absorption loss (SE_A_) range were 14.75–11.73 dB and 43.38–20.01 dB, respectively (Table S2). Further, SE_R_ was high in GNMC with 14.75 dB of maximum while exhibited maximum absorption of 26.97 dB. Among fabricated fabric, absorption played a major role in EMI shielding.

The mechanism of GNMC can be explained. according to structure that the MXene film and graphene nanoplates reflects incident rays caused by moving charges while internal hollow structure, free carriers and layered structure of MXene caused multiple reflection and scattering within the core, finally leads to absorption [77,78]. Further, the highest EMI shielding of MGNC arises owing to its physical nature, i.e., lowest sheet resistance, high resistivity, internal pores, and thickness (Figure 6b). Among the fabricated MC composites, GNOMC shows the highest EMI shielding in S band region owing to the planer nature caused by the functional group derived by means of etching [79]. Even though, in X band region, GNMC, MGNMC and rGNOMC exhibited higher EMI SE compare with GNO as they possessed relatively higher reflection and absorption loss (Figure 7b–d and Table S2). This can be further correlated with cross section of fabric that made cores and randomly arrange GN, rGNO and MXene flake caused absorption (Figure 7b–d and Table S2). In addition, GNO exhibited high EMI shielding in S band region which is due to the high conductivity and in X band region its EMI SE decrease significantly owing to dielectric response rather than electron mobility [80]. Further, planar structure of GNOMC diminished absorption (Figure 7b–d and Table S2). Formation of functional groups promotes interfacial touching of the flake-created planner surface with higher conductivity (Figure 1c and Figure 7). GNMC and MGNMC display similar EMI shielding values. However, MXene-graphene foam exhibits good EMI shielding which can be explained by the fact that the coating of hydrophilic MXene colloidal solution and the hydrophobic GN-PVDF polymer coating on the carbon fiber are limited owing to the adhesion between MXene and graphene. Thus, interfacial electron transfer is minimized owing to the improper arrangement of GN and MXene flake increasing the surface resistance (Figure 7). Hence, the reflection was low for all the composite (Figure 7b–d and Table S2). The specific EMI shielding effectiveness (SSE) of MC, GNMC-single, GNMC, GNOMC, rGNOMC, MGNMC, MGNC, and MGNOC is 381.5, 452.73, 394.91, 189.90, 183.8, 185.3, 46.4, and 56.18 dB·cm^3^·g^−1^, respectively; the single GN-coated composite shows the highest SSE. Furthermore, SSE range of all the composite in X band region was 449.95–68.05 dB·cm^3^·g^−1^ (Table S2). Of the fabricated single-coated composites, GNMC shows the highest absolute EMI shielding effectiveness (SSE/t) of 35,369.82 dB·cm^3^·g^−1^, whereas MC, GNMC, GNOMC, rGNOMC, MGNMC, MGNC, and MGNOC exhibit values of 30,039, 10,914, 11,275.78, 9649.42, 1324.29, and 2311.83 dB·cm^3^·g^−1^, respectively in S band region while SSE/t of composite in X band region was 35428.4–1944.3 dB·cm^3^·g^−1^ in which MGNC displayed lowest SSE/t (Table S2). Further, the thickness influences the EMI shielding. Reducing the amount of PVDF increases SSE/t (PVDF (1 g·L^−1^) and GN (3 g·L^−1^) in DMF, yielding 31,095.13 dB·cm^2^·g^−1^ in S band region). According to reported data, PVDF exhibits an EMI shielding effectiveness of approximately 1.1 dB, which is not an effective barrier against electromagnetic radiation compared with carbon-based PVDF composites [48].

Most of the carbon base composite reported showed lower EMI shielding compared to the composite produced and thickness proportional to EMI SE and increasing graphene loading increase the EMI SE. However, in each case, equal amount of dispersed solutions was utilized. Thus, in this case, not only component loading but also structural feature of composite affect EMI SE (Table S1 and Figure 1, Figure 7e and Figure S2) [77,81,82]. Further, the MXene based composite with less thickness generate relatively good EMI SE compare with other composite reported (Figure 7e) and the Al and Cu foil show exceptional EMI shielding of approximately 70 dB (~10 μm). Gonzalez et al. reported that the reflection from CNT and graphene is approximately 10 dB with an absorption of 20 dB. At the same time, ultrathin graphene-based composites have also shown a lower reflection of approximately 10 dB [75,83,84,85]. According to Zhao et al. the EMI shielding of the PVDF/graphene composite was 22.58 dB at a thickness 0.1 mm and electrical conductivity of 6.56 × 10^−3^ S·cm^−1^ [40]. Poly (ether imide) (PEI-rGO nanocomposite films exhibited EMI shielding values of approximately 26 dB at a thickness of 0.086 mm [41]. PVDF/graphene quantum dots showed a 31 dB EMI shielding at an 8 GHz frequency. Further, Ag-nanoparticle reinforced PVDF/graphene quantum dots increase EMI shielding (43 dB at 12 GHz) [42]. Hence, the composition, amount, and status of graphene in the composition alter the EMI shielding. In addition, the incorporation of nanoparticles improves the EMI shielding of the graphene composites [40,41,42]. Yuan et al. reported that reduced graphene oxide nano-composite films exhibit EMI shielding of 32 dB with 0.27 mm [85]. Based on the literature reviewed, our study shows excellent EMI shielding effectiveness over a frequency range of 1–3 GHz and 8–12.4 GHz.

### 3.5. Thermal Stability and Thermo Gravimetric Analysis of Composites

**Figure 8:**
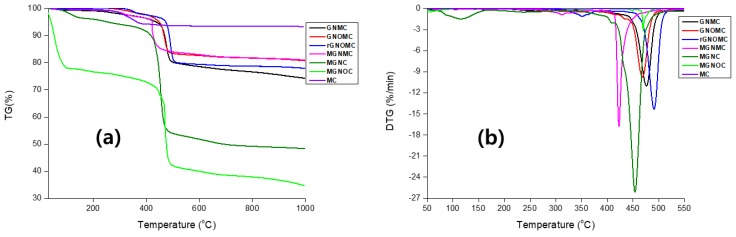
(**a**) TGA and (**b**) DTG curves of composites.

Thermal stability studies were carried out using well-known thermogravimetric analysis (TGA) and differential thermal analysis (DTG). The temperature range was maintained from room temperature to 1000 °C with a heating rate of 10 °C·min^−1^, and during the TGA and DTG analysis, the Al_2_O_3_ crucible and nitrogen environment were maintained. The mass loss and enthalpy changes were investigated using TGA and DTG, respectively. All of the samples exhibit outstanding stability over a higher temperature range (Figure 8a). Swift degradation of all of the composites occurred about 375 °C to 500 °C, which is higher than that of MC, which exhibits a 5% weight loss between 280 °C and 400 °C [7]. Further, MGNOC and MGNC exhibit a 65% and 52% weight loss, respectively, whereas the MC-based composites exhibit a loss of 20% in the aforementioned temperature range. These composites (MGNOC and MGNC) show a higher weight loss than MC (6.5%) [7]. This is due to the introduction of a polymer binder (PVDF and PAA) and GN/GNO to the composite [60,86]. The weight-loss temperature of pristine PVDF and graphene are approximately 400 and 200 °C, respectively [87,88,89,90,91]. We noticed that all of the fabrics exhibit similar behavior below 400 and above 500 °C. All of the fabric shows a minimal weight loss (~20%) which is due to the introduction of graphene species and MXene (Table 2). In addition, the thermal stability of the composites can be altered by amount filler loading, types of polymers used, environment of experience, exposure temperature and duration of exposure of the composites. Presence of oxygen environment burn both polymer, MXene and graphene [92]. The MC had the minimum temperature of the degradation was 40 °C and 174 °C. This trend changed after introduction of graphene (Table 2). Though, minimum degradation temperature of the all composite bellow 300 ℃ that is intermediate temperature of graphene and PVDF. The introduction of the oxygen on graphene increase the weight loss considerably (Table 2). On the whole, PVDF, graphene, MXene film possessed low thermal stability compare with carbon fabric-based composites. Hence, the carbon fabric induces the thermal stability of the composite [7]. MGNC and MGNOC lost 5% and 20% weight at approximately 100 °C which was due to the water loss, and then both constrained degradations up to 400 °C. In addition, above 400 °C, MGNC and MGNOC exhibit a 50% and 65% weight loss, respectively. The DTG curve of the composites shows endothermic peaks at different positions. GNMC, GNOMC, rGNOMC, MGNMC, MGNC, and MGNOC show prominent peaks at 476.9, 468.7, 490.5, 422.4, 453.1, and 469.33 °C, respectively (Figure 8b). This indicated where rapid weight loss occurred. In addition, MC exhibits a broad endothermic peak in the range of 243–390 °C Hence, the stability of the composite dramatically increases with the coating process. The introduction of MXene minimizes the degradation of the composite, which means that all of the MXene-based composites show a low thermal stability. Further, GNOMC and rGNOMC show another endo-thermic peak at 350 °C, which is more intense in rGNO than in GNO. The introduction of GNO and rGNO generates new peaks where they were absent in GN, and a similar peak is observed at 313 °C, which shifts to a lower temperature owing to the presence of MXene. MGNC and MGNOC exhibit the same endo-thermic peaks at 117 °C owing to the thermal conductivity oGN/PVDF, internal pores, LiCl, and PAA. The TG curve supports this statement [76]. Finally, the rGO-based composite displays a higher thermal stability than the other composites fabricated.

## 4. Conclusions

Spray-coated composites and solvent casting films were successfully fabricated with high flexibility, low apparent density (~0.77 to 0.081 g·cm^−3^) and low thickness (0.0120–0.0350 cm). The fabricated composites exhibited an uppermost contact angle of 126° and the range of wetting energy of all of the composites was −42.82 to 14.89 mN·m^−1^. Thus, graphene-based constitutions improve the hydrophobicity. The surface-coated MXene and graphene oxide minimized the sheet resistance and showed a high conductivity of 13.68 S·cm^−1^ with a sheet resistance of 3.1 Ω·sq^−1^. The MXene-graphene-PVDF composition improved the thermal stability and constrained the dramatic weight changes up to 400 °C. The flat stack-like composition displayed an excellent EMI shielding of 41 dB (99.99% efficiency) in S band while exhibited maximum EMI shielding of GNMC 53.8 (99.999%) with reflection of 13.10 dB and absorption of 43.38 dB (Figure 7 and Table S2). and the size of the pore comparatively advanced the property of EMI shielding. The single-coated graphene fabric showed an outstanding absolute shielding effectiveness of 35,369.82 dB·cm^2^·g^−1^. Hence, the composites with high EMI SEs and that are hydrophobic in nature can be applied in various applications such as aeronautics, locators, air travel, mobile phones, handy electronics, and military application.

## Figures and Tables

**Table 1:** Atomic percentages of Ti_3_C_2_T_X_, GN, GNO, rGNO, and composites from XPS analysis.

Elements	C1s (%)	O1s (%)	F1s (%)	Ti (%)	S (%)	N (%)	Si (%)	Cl (%)
MXene	20.54	14.86	58.27	6.32	-	-	-	-
MC	89.54	8.8	-	-	-	1.16	0.51	-
GNMC	81.38	2.46	15.31	-	-	0.85	-	-
GNOMC	73.34	8.16	15.02	-	-	0.63	2.85	-
rGNOMC	75.49	7.66	1.71	-	-	-	-	-
MGNC	56.55	33.69	2.41	3.48	-	1.55	-	2.34
GN	95.42	4.07	-	-	0.52	-	-	-
GNO	92.49	7.51	-	-	-	-	-	-
rGNO	93.12	6.88	-	-	-	-	-	-

**Table 2:** Comparison of mass changes of composites.

No.	Composites	Rapid Change Range (°C)	Rapid Mass Change (%)	Whole Mass Change (%)	Degradation Starting Temperature (°C)
1	GNMC	425–505	15.5	26.0	245.0
2	GNOMC	435–500	11.6	19.5	245.0
3	rGNOMC	460–510	16.1	22.2	265.0
4	MGNMC	420–510	12.0	19.1	275.0
5	MGNC	373–490	38.6	52.1	78.5
6	MGNOC	35–75	22.1	65.5	35.0
		375–510	32.6		75.5
7	MC	175–570	6.2	6.5	40.0
					174.0

## Supplementary Materials

The following are available online at http://www.mdpi.com/1996-1944/11/10/1803/s1, Figure S1: EMI shielding sample loading; Figure S2: Cross section of SEM image; Figure S3: EDX and mapping; Figure S4: Normalized curve of Raman spectrum; Table S1: Comparison of EMI SE with thickness; Table S2: Comparison of maximum (MAX), minimum (MINI), average (AVE) shielding, SSE and SSE/t of composite in each case.
