# Supplementary material for: Electromagnetic Shielding by MXene-Graphene-PVDF Composite with Hydrophobic, Lightweight and Flexible Graphene Coated Fabric
Source: Materials (Basel). 2018 Sep 22;11(10):1803. doi: 10.3390/ma11101803 (PMC6213747; doi:10.3390/ma11101803)
Supplement: Supplementary file 1 [file materials-11-01803-s001.pdf]

materials

MDPI

Supplementary information

Electromagnetic Shielding by MXene-Graphene-PVDF Composite with Hydrophobic, Lightweight and Flexible Graphene Coated Fabric

Electrical Conductivity

The electrical conductivity reciprocally changes with resistance, decreased with the coating process. The resistance of a material can be calculated as [1]

$$R = \varrho \frac{L}{A} = \varrho \frac{L}{Wt} \quad (1)$$

Where, R is the material resistance, ϱ is the resistivity, A is the cross-sectional area, and L is the length. The cross-sectional area can be separated into the width (W) and the sheet thickness (t).

$$R = \frac{\rho L}{tW} = R_s \frac{L}{W} \quad (2)$$

Where, R_s—Sheet resistance

If the film thickness (t) is known, t and R_s can be multiplied to obtain the bulk resistivity ρ (in Ω·cm):

$$\rho = R_s.\ t \quad (3)$$

The reciprocal of the resistivity is the conductivity of the material. $\sigma = 1/\rho$ where, the conductivity of the material can be give as,

$$\sigma = (R_s.\ t)^{-1} \quad (4)$$

The conductivity of the material was calculated according to the above Equation.

The electromagnetic interference shielding effectiveness (EMI SE), is a measure of blocking electromagnetic waves (EMW).

EMI SE is experimentally defined as the logarithmic ratio of incoming power (P_I) to transmitted power (PT) [2] that is measured in decibel (dB),

$$SE\ (dB) = \log_{10}(P_I/P_t) \quad (5)$$

When an EM radiation is incident on shielding film, the incident power (P_I) can be expressed the added combination of the reflected power (P_R), absorbed power (P_A), and transmitted power (P_T)

$$P_I = P_R + P_A + P_T \quad (6)$$

For the intensity (I) it is,

$$I_O = I_R + I_A + I_T \quad (7)$$

Specific Shielding Effectiveness (SSE- dB·cm^3·g^{-1}) is mathematically expressed. Here, SSE is calculated dividing the EMI SE by the density of material (ρ).

$$SSE = \frac{EMI\ SE}{density} \quad (8)$$

SSE gives a more accurate account on EMI SE compare to ρ of the material where, thinner material might be having higher EMI SE [1–7].

The SSE does not give thickness-based information while absolute effectiveness (SSE/t-dB·cm^2·g^{-1}) is used to evaluate the relationship between SSE and thickness.

$$SSE/t = \frac{SSE}{t} \quad (9)$$

EMI Shielding Measurement

The Figure S1 showed instrumentation and sample shape with size. The outer diameter of the sample was 13.3 cm and middle had opening with 3.3 cm diameter. The samples were cut manually and placed in the circular region exhibited in Figure S1b for the analysis.

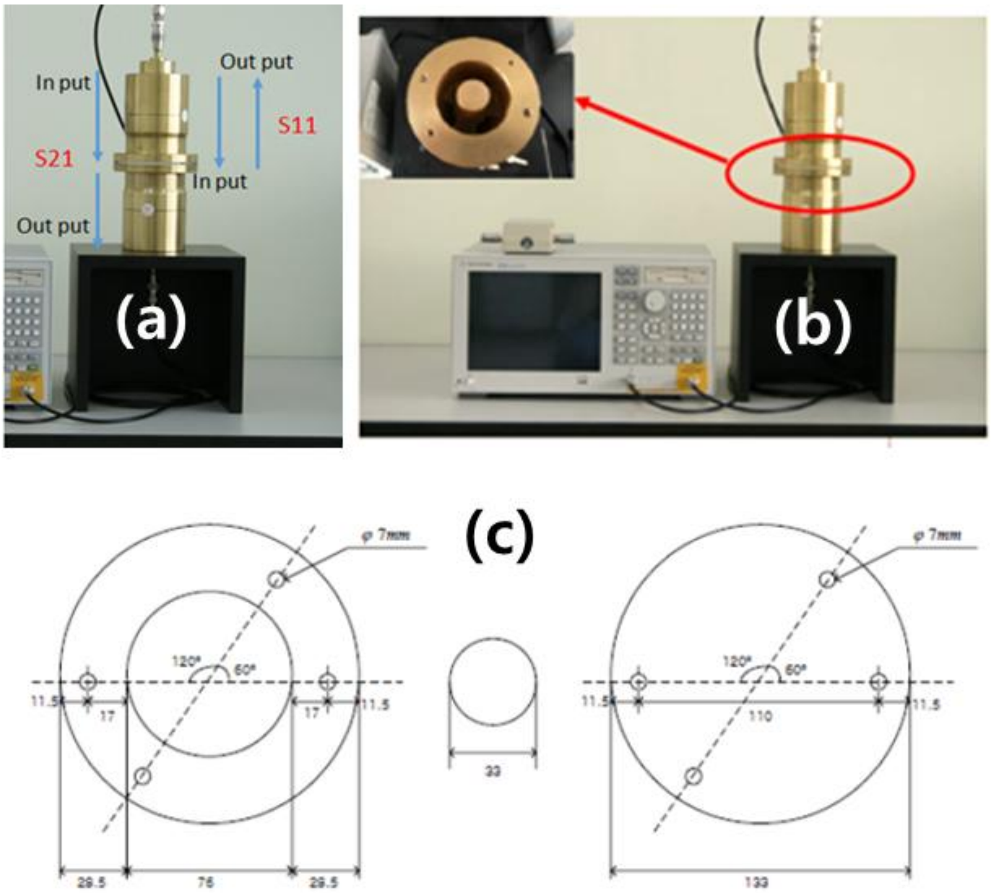

Figure S1. EMI shielding sample loading (**a**) sample loading unit, (**b**) cross section of sample loading side and (**c**) format of sample.

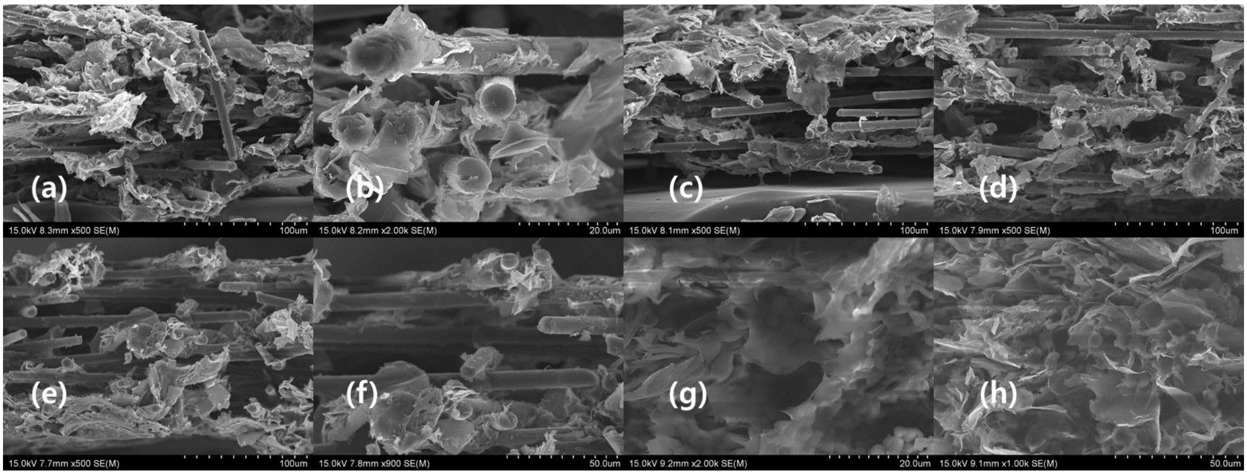

Figure S2. Cross section of SEM image (**a**) GNMC (×500), (**b**) GNMC (×2000), (**c**) GNOMC (×500), (**d**) rGNOMC (×500), (**e**) MGNMC (×500), (**f**) MGNMC (×900), (**g**) MGNC (×2000) and (**h**) MGNOC (×1000).

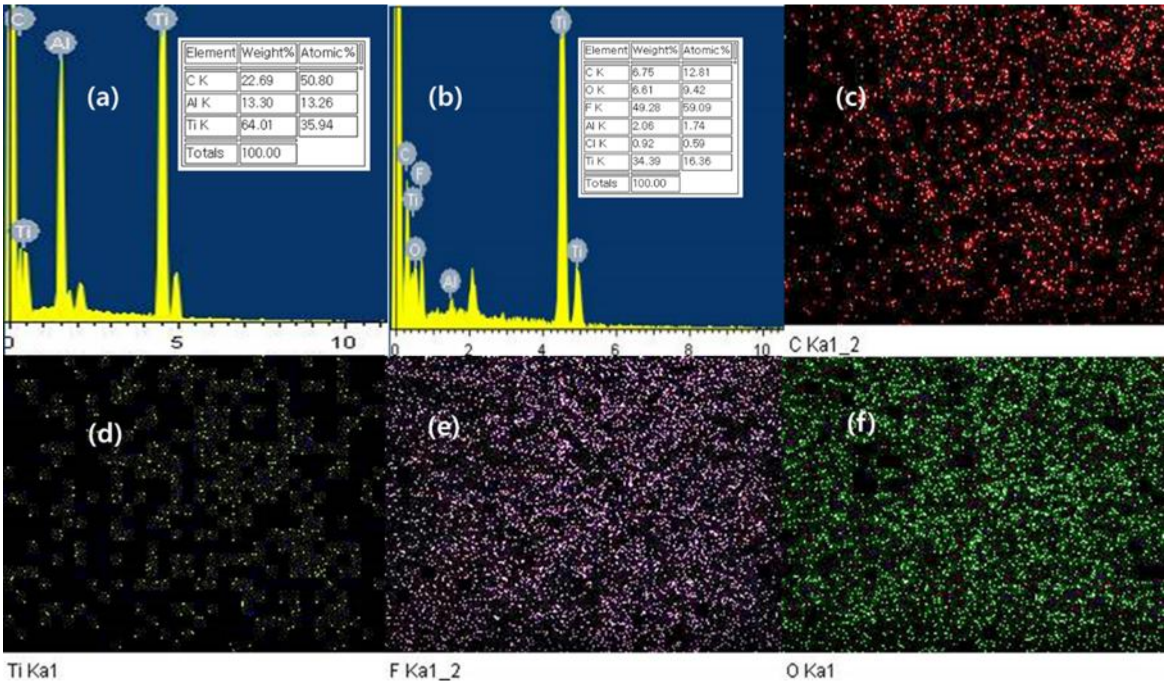

Figure S3. EDX of (**a**) Ti_3AlC_2, (**b**) $Ti_3C_2T_x$; mapping of MGNC (**c**) C, (**d**) Ti, (**e**) F, and (**f**) O.

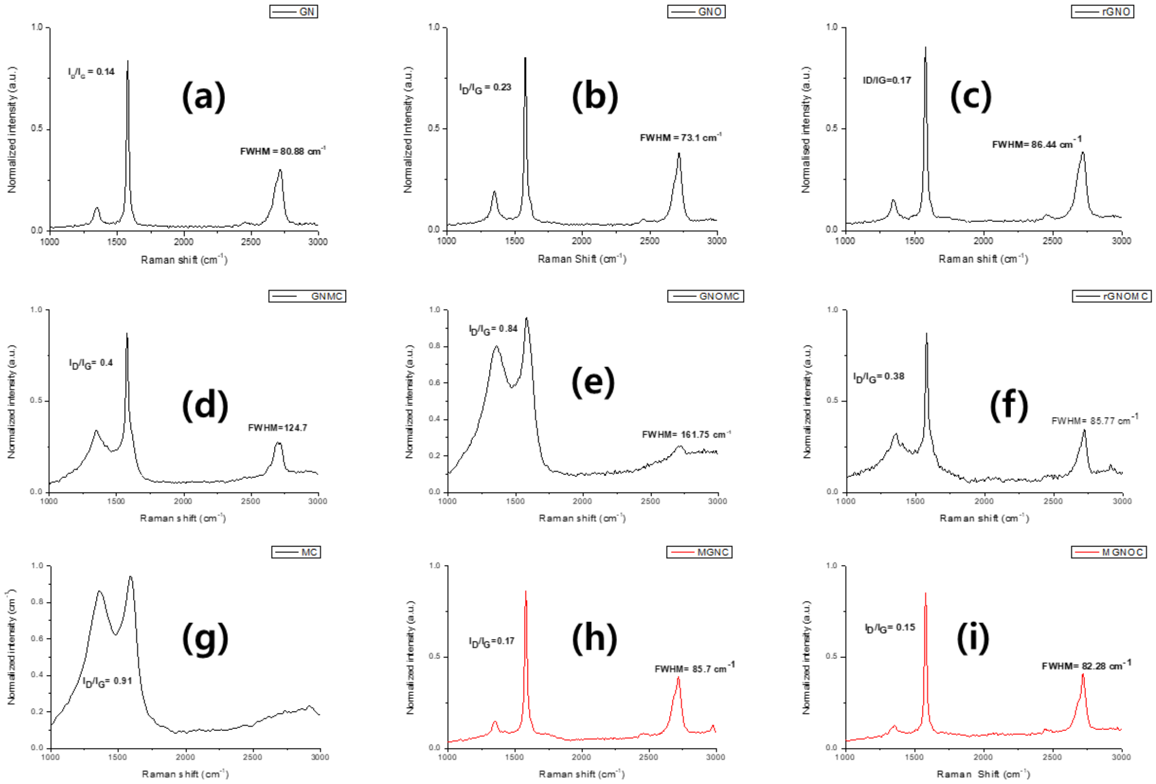

Figure S4. Normalized curve of Raman spectrum of (**a**) GN, (**b**) GNO, (**c**) rGNO, (**d**) GNMC, (**e**) GNOMC, (**f**) rGNOMC, (**g**) MC, (**h**) MGNC and (**i**) MGNOC

Table S1. Comparison of EMI SE with thickness.

No.	Compositon	Filler	t(mm)	SE (dB)	SSE(dB·cm³·g⁻¹)	SSE/t (dB·cm²·g⁻¹)	Ref.
1	GN/Fe_3O_4	0	0.3	24	31	1033	7
2	MWCNT	PC	2.1	39	34.5	154	8
3	CB	EPDM	2	18	30.3	15.1	9
4	CB	ABS	1.1	20	20.9	190	10
5	MWCNT	PS	2	30	57	285	11
6	CNT	POLYM	3.5	80	*	*	12
7	Cu foil	0	0.01	70	7.8	7812	2
8	Al foil	0	0.008	66	24.4	30555	2
9	stainless steel	0	4	89	11	27.5	13
10	MWCNT/NCF	0	0.138	28.22	486.54	35256	14
11	NCF-20 g/m²	0	0.127	25.56	381.50	30039	14
12	Cu bulk	0	3.1	90	10	32.3	13
13	Ni fiber	PES	2.85	58	31	108.7	13
14	Mxene	PET	0.045	92	138.735	30,830	2
15	MXene	cellusolse	0.047	25.8	12.44	2647	15
16	Mxene/foam	0	0.06	70	*	*	16
17	Mxene	parafine	1	76.1	*	*	17
18	Graphene	PVDF/MWCNTs (PF/CNT)	2	28.5	*	*	18
19	rGO	$PbTiO_3$/PEDOT	2.5	51.94	*	*	19
20	CNT	polypropylene	2.2	48.3	*	*	20
21	Graphene nanoplate	B_4C	2	38	*	*	21
22	Graphene	PVDF	3	37.4	*	*	22
23	Large size graphene (LG)	Doping by iodine	0.0125	52.2	*	*	23
24	Expanded graphite (EG)	Large Flexible Graphene	0.043	48.3	*	*	24
25	GNP	PBAT	1	14	*	*	25
26	Porous Fe_3O_4	C	4.27	54.6	*	*	26
27	CF-30 g/m²	0	0.219	27.14	381.5	30039	This work
28	MGNC-S band	polymer	0.35	43.2	46.4	1324.29	1-3 GHz
29	MGNC-X band	polymer	0.35	53.88			This work 8-12.25 GHz

* Sign indicates that the values were impossible to calculate or not available enough data to calculate. Densities of CF and MGNC were 0.146 g cm^{-3} and 0.77 g cm^{-3} respectively.

Table S2. Comparion of maximum (MAX), minimum (MINI), average (AVE) shielding,SSE and SSE/t of composite in each case.

Bands	Type of SE (dB)		MC	GNMC	GNOMC	rGNOMC	MGNMC	MGNC	MGNOC
X- Band	EMI SE	MAX	31.73	41.30	37.88	39.28	38.99	53.89	44.10
		MINI	29.45	39.55	35.88	37.40	37.23	51.64	41.99
		AVE	30.15	39.99	36.40	37.89	37.66	52.40	42.60
	SE_R	MAX	11.73	14.75	13.45	14.37	13.50	13.10	13.62
		MINI	10.31	12.64	11.57	12.59	11.50	10.43	11.84
		AVE	10.77	13.40	12.25	13.20	12.22	11.46	12.46
	SEA	MAX	20.01	26.97	24.46	24.95	25.75	43.38	30.51
		MINI	19.10	26.36	23.99	24.58	25.28	38.73	29.95
		AVE	19.37	26.60	24.15	24.70	25.44	40.94	30.13
S- Band	EMI SE	MAX	28.48	35.30	36.16	34.55	35.20	40.79	35.91
		MINI	23.23	28.44	29.75	28.44	28.76	30.94	23.14
		AVE	25.56	31.99	32.66	31.43	31.87	35.69	32.86
X- Band	SSE ($dBcm^3g^{-1}$)		449.95	268.38	211.64	221.59	218.98	68.05	72.81
	SSE/t ($dBcm^2g^{-1}$)		35428.4	14051.2	12163.1	13594.2	11405.3	1944.3	2996.2
S- Band	SSE ($dBcm^3g^{-1}$)		381.5	394.9	189.9	183.8	185.3	46.4	56.18
	SSE/t ($dBcm^2g^{-1}$)		30039	35369.82	10914	11275.8	9649.42	1324.3	2311.83

References

1. Ameli, A.; Nofar, M.; Wang, S.; Park, C.B. Lightweight polypropylene/stainless-steel fiber composite foams with low percolation for efficient electromagnetic interference shielding. *ACS Appl. Mater. Interfaces* **2014**, *6*, 11091–11100.
2. Shahzad, F.; Alhabeb, M.; Hatter, C.B.; Anasori, B.; Hong, S.M.; Koo, C.M.; Gogotsi, Y. Electromagnetic interference shielding with 2D transition metal carbides (MXenes). *Science* **2016**, *353*, 1137–1140.
3. Bian, X.M.; Liu, L.; Li, H.B.; Wang, C.Y.; Xie, Q.; Zhao, Q.L.; Hou, Z.L. Construction of three-dimensional graphene interfaces into carbon fiber textiles for increasing deposition of nickel nanoparticles: Flexible hierarchical magnetic textile composites for strong electromagnetic shielding. *Nanotechnology* **2016**, *28*, 045710.
4. Yan, D.X; Pang, H.; Li, B.; Vajtai, R.; Xu, L.; Ren, P.G.; Li, Z.M. Structured reduced graphene oxide/polymer composites for ultra-efficient electromagnetic interference shielding. *Adv. Funct. Mater.* **2015**, *25*, 559–566.
5. Zeng, Z.; Jin, H.; Chen, M.; Li, W.; Zhou, L.; Zhang, Z. Lightweight and anisotropic porous MWCNT/WPU composites for ultrahigh performance electromagnetic interference shielding. *Adv. Funct. Mater*. **2016**, *26*, 303–310.
6. Han, Z.; Fina, A. Thermal conductivity of carbon nanotubes and their polymer nanocomposites: A review. *Prog. Polym. Sci*. **2011**, *36*, 914–944.
7. Agnihotri, N.; Chakrabarti, K.; De, A.; Highly efficient electromagnetic interference shielding using graphite nanoplatelet/poly(3,4-ethylenedioxythiophene)-poly(styrenesulfonate) composites with enhanced thermal conductivity. *RSC Adv.* **2015**, *5*, 43765–43771.

8. Pande, S.; Chaudhary, A.; Patel, D.; Singh, B.P.; Mathur, R.B. Mechanical and electrical properties of multiwall carbon nanotube/polycarbonate composites for electrostatic discharge and electromagnetic interference shielding applications. *RSC Adv.* **2014**, *4*, 13839.
9. Ghosh, P.; Chakrabarti, A. Conducting carbon black filled EVA vulcanizates: Assessment of dependence of physical and mechanical properties and conducting character on variation of filler loading. *J. Polym. Mater.* **2000**, *17*, 291–304.
10. Al-Saleh, M.H.; Saadeh, W.H.; Sundararaj, U. EMI shielding effectiveness of carbon based nanostructured polymeric materials: A comparative study. *Carbon* **2013**, *60*, 146–156.
11. Arjmand, M.; Apperley, T.; Okoniewski, M.; Sundararaj, U. Comparative study of electromagnetic interference shielding properties of injection molded versus compression molded multi-walled carbon nanotube/polystyrene composites. *Carbon* **2012**, *50*, 5126–5134.
12. Micheli, D.; Vricella, A.; Pastore, R.; Delfini, A.; Giusti, A.; Albano, M.; Primiani, V.M. Ballistic and electromagnetic shielding behaviour of multifunctional Kevlar fiber reinforced epoxy composites modified by carbon nanotubes. *Carbon* **2016**, *104*, 141–156.
13. Shui, X.; Chung, D.D.L. Nickel filament polymer-matrix composites with low surface impedance and high electromagnetic interference shielding effectiveness. *J. Electron. Mater.* **1997**, *26*, 928–934.
14. Pothupitiya Gamage, S.J., Yang, K., Braveenth, R., Raagulan, K., Kim, H.S., Lee, Y.S., Yang, C.M., Moon, J.J.; Chai, K.Y. MWCNT coated free-standing carbon fiber fabric for enhanced performance in EMI shielding with a higher absolute EMI SE. *Materials* **2017**, *10*, 1350.
15. Cao, W.T.; Chen, F.F.; Zhu, Y.J.; Zhang, Y.G.; Jiang, Y.Y.; Ma, M.G.; Chen, F. Binary Strengthening and Toughening of MXene/Cellulose Nanofiber Composite Paper with Nacre-Inspired Structure and Superior Electromagnetic Interference Shielding Properties. *ACS Nano* **2018**, *12*, 4583–4593.
16. Liu, J.; Zhang, H.B.; Sun, R.; Liu, Y.; Liu, Z.; Zhou, A.; Yu, Z.Z. Hydrophobic, Flexible, and Lightweight MXene Foams for High-Performance Electromagnetic-Interference Shielding. *Adv. Mater.* **2017**, *29*, 1702367.
17. Han, M.; Yin, X.; Wu, H.; Hou, Z.; Song, C.; Li, X.; Zhang, L.; Cheng, L. Ti3C2 MXenes with modified surface for high-performance electromagnetic absorption and shielding in the X-band. *ACS Appl. Mater. Interfaces* **2016**, 8, 21011–21019.
18. Ma, X.; Shen, B.; Zhang, L.; Liu, Y.; Zhai, W.; Zheng, W. Porous superhydrophobic polymer/carbon composites for lightweight and self-cleaning EMI shielding application. *Compos. Sci. Technol.* **2018**, *158*, 86–93.
19. Dalal, J.; Lather, S.; Gupta, A.; Dahiya, S.; Maan, A.S.; Singh, K.; Dhawan, S.K.; Ohlan, A. EMI shielding properties of laminated graphene and PbTiO3 reinforced poly (3,4-ethylenedioxythiophene) nanocomposites. *Compos. Sci. Technol.* **2018**, *165*, 222–230.
20. Wu, H.Y.; Jia, L.C.; Yan, D.X.; Gao, J.F.; Zhang, X.P.; Ren, P.G.; Li, Z.M. Simultaneously improved electromagnetic interference shielding and mechanical performance of segregated carbon nanotube/polypropylene composite via solid phase molding. *Compos. Sci. Technol.* **2018**, *156*, 87–94.
21. Tan, Y.; Luo, H.; Zhang, H.; Zhou, X.; Peng, S. Lightweight graphene nanoplatelet/boron carbide composite with high EMI shielding effectiveness. *AIP Adv.* **2016**, *6*, 035208.
22. Zhao, C.; Hamidinejad, M.; Wang, C.; Li, R.; Wang, S.; Yasamin, K.; Park, C.B. Incorporating a microcellular structure into PVDF/graphene-nanoplatelet composites to tune their electrical conductivity and electromagnetic interference shielding properties. *J. Mater. Chem. C* **2018**, doi:10.1039/C8TC03714K.
23. Wan, Y.J.; Zhu, P.L.; Yu, S.H.; Sun, R.; Wong, C.P.; Liao, W.H., Graphene paper for exceptional EMI shielding performance using large-sized graphene oxide sheets and doping strategy. *Carbon* **2017**, *122*, 74–81.
24. Liu, Y.; Zeng, J.; Han, D.; Wu, K.; Yu, B.; Chai, S.; Chen, F.; Fu, Q. Graphene enhanced flexible expanded graphite film with high electric, thermal conductivities and EMI shielding at low content. *Carbon* **2018**, *133*, 435–445.
25. Kashi, S.; Hadigheh, S.A.; Varley, R. Microwave Attenuation of Graphene Modified Thermoplastic Poly (Butylene adipate-co-terephthalate) Nanocomposites. *Polymers* **2018**, *10*, 582.
26. Wu, N.; Liu, C.; Xu, D.; Liu, J.; Liu, W.; Shao, Q.; Guo, Z. Enhanced electromagnetic wave absorption of three-dimensional porous Fe3O4/C composite flowers. *ACS Sustain. Chem. Eng.* **2018**, *6*, 12471–12480.
